# Epidemiology of multiple sclerosis in Qom: Demographic study in Iran

**Published:** 2013

**Authors:** Saeed Rezaali, Ahad Khalilnezhad, Abdorreza Naser Moghadasi, Samira Chaibakhsh, Mohammad Ali Sahraian

**Affiliations:** 1 Researcher, MS Research Center, Neuroscience institute, Sina Hospital, Tehran University of Medical Sciences AND Tehran Medical Branch, Islamic Azad University, Tehran, Iran; 2 Department of Immunology, School of Medical, Shahid Beheshti University of Medical Sciences, Tehran, Iran; 3 Neurologist, Researcher, Department of Neurology AND MS Research Center, Neuroscience institute, Sina Hospital, Tehran University of Medical Sciences, Tehran, Iran; 4 Department of Biostatistics, Shahid Beheshti University of Medical Sciences, Tehran, Iran; 5 Associate Professor, Department of Neurology AND MS Research Center, Neuroscience institute, Sina Hospital, Tehran University of Medical Sciences, Tehran, Iran

**Keywords:** Demography, Epidemiology, Iran, Multiple Sclerosis, Prevalence

## Abstract

**Background:**

Recent studies have demonstrated controversial results and somewhat increased frequency of multiple sclerosis (MS). We reevaluated the files of MS patients from Qom Province of Iran in order to investigate the epidemiology of the disease.

**Methods:**

Demographic and clinical records of 592 MS patients were reviewed, which included; age, sex, date of birth, marital and occupation status, presenting symptoms, time of onset, type and family history of MS, and history of autoimmune or other diseases.

**Results:**

At the time of our study, 11 patients had died, and 581 were alive with a total female-to-male ratio of 3.4. The mean age of onset of the disease was 34.25 ± 9.01 for all the patients. 11.2% of patients had positive family history of MS. The majority of patients (80.1%) showed relapsing-remitting (RR) pattern. The prevalence of MS was calculated as 50.4/100000 for Qom.

**Conclusion:**

Qom is located within a high risk zone of MS. Although we found evidences about the role of environmental factors, geographical distribution, and etcetera, many more studies need to be performed in this respect.

## Introduction

Multiple sclerosis (MS) is the most common chronic inflammatory demyelinating disease of the central nervous system that mainly affects young adults and may cause significant disability.^1, 2^ It is postulated that an aberrant immune response to myelin antigens may be implicated in the pathogenesis of MS.^3, 4^ However, the actual cause of the disease remains to be elucidated.

Evidences from studies of different populations over various geographical areas suggested that a complicated interaction of genetic and environmental factors may be involved in the development of MS.^5, 6^ A theory that could be further supported by highly informative epidemiologic studies.^7^ Indeed, it is believed that achieving a good knowledge of the epidemiology of MS gives new insights into the underlying causes of the disease.

Although many studies have shown significant variance in the prevalence of MS within diverse populations and different geographic areas, a unique geographic distribution for MS has been suggested by many investigators. In addition, an increasing gradient with latitude, has been illustrated for the prevalence of MS within temperate regions, and a generally high prevalence of the disease is documented for the areas located farther away from the equator.^8–10^ However, some contradictory data have been reported in areas possessing the same latitude and even in the same countries.^11–14^

Based on the division of Kurtzke, Iran lies within a low risk zone (less than 5/100000) of MS prevalence.^8, 9^ Although little is done about the epidemiology of MS in Iran, recent surveys in different parts of the country have demonstrated diverse results and somewhat, increased frequency of the disease from low to medium.^15–19^

In this study, we reevaluated the files of 581 patients from Qom province in order to investigate the epidemiology of MS in this province.

## Materials and Methods

### Study Area

This study was conducted during February 2011 to August 2011 in Qom Province located in the center of Iran, within the latitudes and the longitudes, 34.15°-35.15° N and 50.30°-51.30° E, respectively. It is 11237 km^2^ in area, coverings 0.8.6% of the total area of the country, and was a part of Tehran province until 1995. Since Qom province is situated beside an arid region, it has a dry, desert, and semi-desert climate with inadequate rainfall and low humidity.^20^

### Population at Risk

According to the 2011 census, the total population of Qom province was 1151672 people among which 564011 (48.97%) were women and 587661 (51.03%) men, and out of which 1095871 (95.15%) lived in urban areas, 55798 (4.85%) in rural vicinities, and 3 in an undisclosed place.^21^

### Data Collection

Demographic and clinical records of 592 MS patients of Qom province were obtained from Iranian MS Society (IMSS) and mostly from the MS Society of Qom (MSQ), both non-governmental organizations established in 1999 and 2003, respectively, and reviewed. As some habitants of Qom suffering from MS travel to Tehran for treatment, their documents were accessible through IMSS. In the cases of insufficient information or missing data, if possible, phone contacts with the patients were made; otherwise, they were excluded from the study. Note that all the patients included in this study had been living in Qom for the last 10 years before the onset of the disease.

Since MSQ is the unique NGO in Qom for MS patients, and the membership in MSQ and IMSS facilitates the accessibility of some drugs including interferon beta; the patients voluntarily refer to these societies. They are registered by presenting a confirmation letter from the neurologist who has diagnosed the disease based on the worldwide-accepted criteria of Poser et al., and McDonald et al.^22, 23^

Demographic data include age, sex, date of birth, marital and occupational status, number of siblings and children, home address, and smoking status. Clinical information, recorded by a trained general practitioner through an interview with the patient, provides the time of onset, diagnosis and presenting symptoms of the disease, pattern of progression, clinical course, and family history of autoimmune or other diseases, surgeries and etcetera.

### Ethics

To prevent duplications of the data, a single ID number was dedicated to each patient. An informed consent letter was signed by each patient. Moreover, in all stages of the study, the last version of the Declaration of Helsinki was followed by the researcher, and the institutional ethical committee approved the use of the clinical information.

### Statistics

Data analysis was done using the Statistical Package for Social Sciences, (SPSS 17.0, SPSS Production Facility, Chicago, Illinois, USA). Count and percentage of all qualitative variables, and mean and SD of all quantitative variables are presented in tables and figures.

## Results

Among 592 registered patients in MSQ and IMSS, 581 were alive and 11 had died at the time of our investigation; their information was not included in our analysis. Based on the information by the 2011 census that estimated the population of Qom province to be 1151672, and considering the total of 581 MS patients of Qom, the prevalence of the disease was calculated as 50.4/100000 for this province. Unfortunately, as one of the limitations of our study, it was not possible to calculate the incidence of MS disease in Qom.

**Figure 1:**
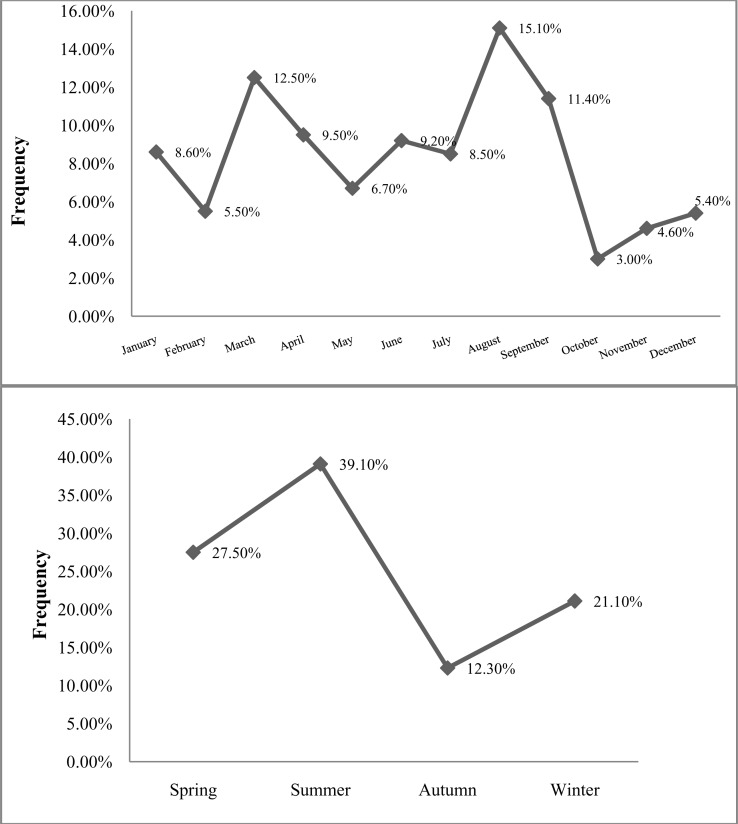
Frequency of MS according to the season and month of birth of the patients from Qom The patients born in summer and in August were more frequent than other seasons and months

Demographic features and some life style factors of the included patients are given in Table 1. The percentage of the women with MS exceeds that of the men, showing a total female-to-male ratio of 3.4. Table 2 summarizes the family history of MS, and other autoimmune disorders, and personal childhood history of infectious (viral) diseases. 11.2% of patients had positive family history of MS among their first-degree relatives, and 19.5% of them hold family history of other autoimmune diseases (Table 2). As illustrated in Figure 1, the patients born in summer and in August were more frequent than other seasons and months, and a minority of the patients was born in autumn and October. However, no significant differences were shown between them.

**Table 1:** Demographic features and some life style factors of MS patients of Qom

Characteristics	Variations n (%)	Frequency n (%)	Total
Gender	Female	449 (77.3)	581
	Male	132 (22.7)	
	Single	124 (22.1)	
Marital status	Married	405 (72.5)	559
	Widowed/Divorced	30 (5.4)	
	Housewife	296 (65.9)	
	Employed	68 (15.1)	
Occupation (women)	Unemployed	56 (12.5)	449
	Student	26 (5.8)	
	Retired	3 (0.7)	
	Employed	73 (57.9)	
Occupation (Men)	Unemployed	45 (35.7)	126
	Student	5 (4.0)	
	Retired	3 (2.4)	
	No immigration	313 (58.7)	
Immigration to Qom	> 15 years	114 (21.4)	533
	< 15 years	106 (19.9)	
Smoking status	Non-smoker	447 (86.1)	519
	Smoker	72 (13.9)	

**Table 2:** Family history of Ms and history of other diseases among MS patients from Qom

Type of disease	Name of disease	Yes n (%)	NO n (%)	Total
	MS	64 (11.2)	508 (88.8)	572
	Hypothyroidism	62 (11.2)	492 (88.8)	554
Family history of Autoimmune diseases	Rheumatoid arthritis	32 (5.8)	522 (94.2)	554
	Hypothyroidism/RA	3 (0.5)	551 (99.5)	554
	SLE	3 (0.5)	551 (99.5)	554
	Regardless of disease name	100 (18.1)	454 (81.9)	554
	Measles	152 (27.2)	406 (72.8)	558
Childhood history of infectious diseases	Rubella	32 (5.8)	523 (94.2)	555
	Mumps	130 (23.5)	423 (76.5)	553
	Chickenpox	305 (54.5)	255 (45.5)	560
	Hepatitis	6 (1.2)	515 (98.8)	521

**Figure 2:**
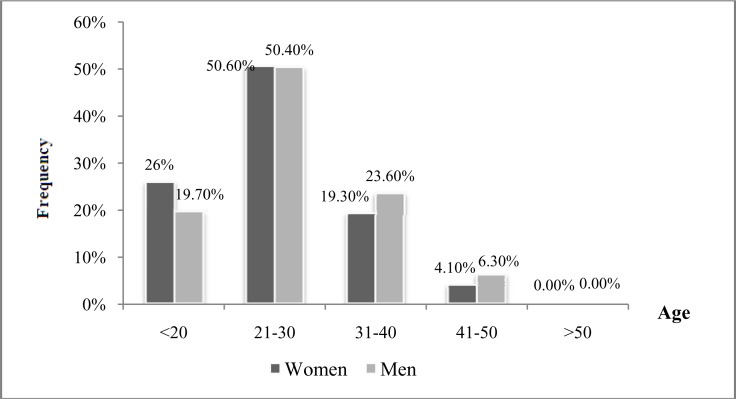
The age of onset of MS among patients from Qom Approximately half of the patients, both women and men, were 21-30 years old when the disease started, while a minority of them was 41-50.

In our study, the first episode of neurological impairment, lasting for 24 hours stated by the patients, was considered as the onset of MS, regardless of the diagnosis of the disease. The mean age of onset of the disease was calculated to be 34.25 ± 9.01 for all the patients, 33.63 ± 8.83 for women, and 36.41 ± 9.34 for men. Figure 2 categorizes the patients in four age groups ranging from bellow 20 year olds up to 50 year olds. As illustrated in this figure, approximately half of the patients, both women and men, were 21-30 years old when the disease started, while a minority of them was 41-50. We conceder the age bellow 16 years as an early-onset age, and above 50 years as late-onset age of MS.^24^ In total, 2.6% of the patients involved in our study appeared to be in the early-onset age and none of the patients were in the late-onset-age category.

**Figure 3:**
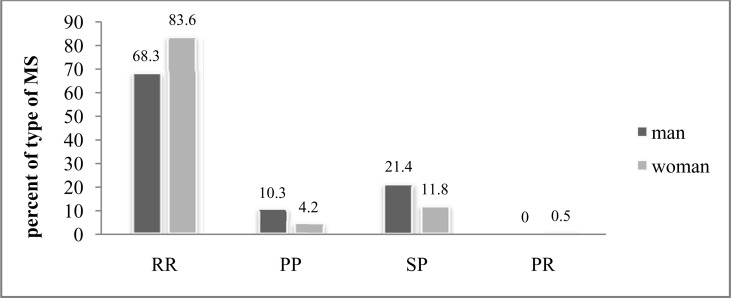
Type of MS among patients from Qom The majority of the patients suffered from relapsing-remitting (RR), both women and men, at the time of registration.

With respect to the type of MS, depicted in Figure 3, the majority of the patients (80.1%) suffered from relapsing-remitting (RR), both women (83.5%) and men (68.3%), at the time of registration. The patients with secondary-progressive (SP) pattern, also appeared with considerable frequency (14.0%); likely more common among men and with a female-to-male ratio of 0.55. According to Table 3, the time interval between the onset of disease and definite MS diagnosis are categorized as less than 6 months (53.1%), from 6 months to 1 year (21.2%), and more than 1 year (25.7%). In addition, with respect to the presenting symptoms of the disease, higher frequencies were calculated for sensory (38.7%), visual (35.9%), and motor (12.7%) signs.

**Table 3:** The time interval between the onset of disease and definite MS diagnosis among patients from Qom

Variation	< 6 months n (%)	6 months-1 year n (%)	> 1 year n (%)	Total
Total	300 (53.1)	120 (21.2)	145 (25.7)	565
Women	235 (53.7)	95 (21.6)	108 (24.7)	438
Men	65 (51.2)	25 (19.7)	37 (29.1)	127
SP	35 (45.5)	17 (22.0)	25 (32.5)	77
RR	251 (56.6)	92 (20.7)	101(22.7)	444
PP	8 (25.8)	9 (29.0)	14 (45.2)	31
PR	2 (100)	0 (00.0)	0 (00.0)	2

SP: secondary-progressive, RR: relapsing-remitting, PP: primary progressive, PR: progressive-relapsing

**Figure 4:**
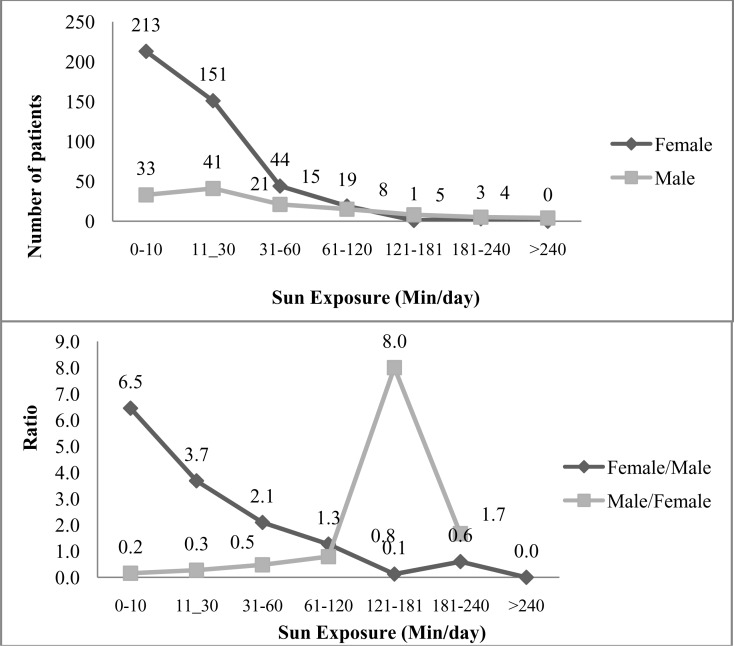
The amount of time of exposure to the sun (minutes/day) among patients from Qom 49.4% and 35.1% of the women, and 25.4% and 32.5% of men were exposed to the sun for 0-10 and 11-30 minutes daily, respectively.

The amount of time of exposure to the sun ranged from 0 to more than 240 minutes per day among the MS patients. 49.4% and 35.1% of the women were exposed to the sun for 0-10 and 11-30 minutes daily, respectively. In contrast, 25.4% of men were exposed for 0-10 minutes, and 32.5% for 11-30 minutes to the sun (Figure 4).

## Discussion

Iran has been previously thought to be located in the low risk zone of MS prevalence.^9, 25^ However, recent epidemiological investigations by Etemadifar et al., and Saadatnia et al. in Isfahan, a province in the center of Iran with a homogeneous racial background, suggested a medium-to-high risk level of MS prevalence in the province.^15, 16^ They reported a prevalence of 35.5/100000 and 43.8/100000, respectively. In addition, recently, our study group in a demographic study of 8000 patients from Tehran, the most populated province of the country with a heterogeneous racial background, confirmed the findings of Etemadifar et al., and Saadatnia et al.^15, 16, 26^ This study presented a prevalence of 51.9/100000 in Iran. More recently, Moghtaderi et al., in an epidemiological study of MS in the southeastern part of Iran, concluded that the incidence rate of MS shows a faster growth rate, compared to previous years.^17^ Furthermore, Hashemilar et al. reported that MS prevalence in North West of Iran lies in the medium frequency range.^18^ Similarly, Jajvandian et al. have described a medium risk area for definite MS in the North East of Iran.^19^

In accordance with all the former studies already mentioned, our present survey among MS patients from Qom province further strenthens the evidences of striking increase in prevalence of MS within the country.^15–17, 19, 26^ Based on our findings, Tehran and Qom have the highest prevalence of MS in comparison with other parts of Iran. Regarding a medium prevalence of MS reported for South East, North West, and North East of Iran; it seems that the distribution of MS does not follow a definite pattern.^17–19^ This together with the results reported from both eastern and western populations may suggest a need to revise the traditional classification of geographic distribution of the disease.^27–31^ However, more investigations must be done.

It is believed that MS is predominant among women compared to men.^14, 32, 33^ In agreement with previouse studies, and according to the results presented by Kalanie et al. and recently by our group, female preponderance has been observed among Iranian MS patients.^26, 33^ This is now further supported by the results of the peresent study, showing a female-to-male ratio of 3.4. Moreover, the results of the present study revealed that the RR type was the predominant pattern of MS, either totally or among the women, which is in accordance with previous reports.^26, 33^ However, we found that SP MS was rather more common than primary progressive (PP) MS, at the time of registration. Moreover, the number of men suffering SP and or PP exceed that of women, that is not in accordance with our previos findings, and findings of Montalban and Rio and Kalanie et al.^26, 33, 34^ They have shown a high frequency of PP, compred to SP, and dominant number of women with PP or SP in comparison with men.

The results of the present study estimated that the mean age of the onset of MS in women is less than men, approximately 3 years. Additionally, 70.8% of the patients were 20-40 years old when the first episode of neurological impairments had occurred, and only 2.6% of the patients faced the early onset of MS. It seems that MS mostly occurs in adults, and the late-onset age, calculated 0.0% in the present study, and the early-onset age, both account for an inconsiderable percentage of the onset-age of the disease among the Iranian population. This is in-line with our previous results and with those of many other investigations.^16, 26, 35, 36^ In addition, regarding gender and type of disease, most of the patients are diagnosed within 6 months after the onset of the disease, which is in agreement with our previous findings.^26^

We found about a three times higher prevalence of MS among married people, both the women and the men, than single patients. In addition, widowed or divorced patients, compared to single patients, appeared to be at a lower risk level. These results are in agreement with our former data from investigating the epidemiology of MS in Tehran.^26^ Unfortunately, as the weakness of our study, we could not involve the spouses of the patients in the study, and we also did not know whether the disease happened before the marriage or after. Moreover, there are few studies concerning the role of marriage status in MS.^37^

It is suggested that in general, individuals with MS have a genetic predisposition to autoimmunity.^38^ Furthermore, autoimmune disease is more common in first-degree relatives of patients with MS; therefore, common genetic susceptibility factors for autoimmunity co-exist with additional disease specific genetic or environmental factors.^39^ Our results indicated that the familial rate among MS patients from Qom province is 11.2%, which is closer to the commonly-accepted rate reported by Compston, and in line with the studies of El-Salem et al., and Ebers et al.^31, 40, 41^ In addition, we found that a considerable percentage of the patients hold a family history of other autoimmune diseases. There are some evidences that viral infections may contribute to the increase in susceptibility to MS in both children and adults; however, some controversial data exist.^42–46^ We observed that a considerable number of the patients had experienced Chickenpox, Measles, and Mumps during their childhood, which may support the association between viral infections and MS; though, this remains to be well-elucidated.

Seasonal effects on risk of MS has been suggested by many studies, describing the patients born in spring with higher frequency of MS births and those born in the autumn with less frequency of MS.^47–50^ However, exceptions have also been reported.^51, 52^ According to the results of the present study, the majority of patients were born in summer and in August and the patients born in autumn and in October appeared with less frequency. Our study shows no significant differences among months and seasons of both of the MS patients. This is in agreement with the studies by Givon et al. and Staples et al.^51, 52^ However, this is in discordance with the study of Bayes et al., who have mentioned the peak season and month to be spring and April, respectively.^48^ However, the month and season of birth needs further research to be considered as a climate-related environmental factor involved in susceptibility to MS. It is thought that exposure to sunlight, due to its role in vitamin D production, is involved in the development of MS.^53–56^ Our findings might support the previous data about the role of the reduction in exposure to sunlight in determining the rate of MS.^53–56^

## Conclusion

According the results of the present study, Qom is located within a high risk zone of MS. In addition, regarding the previous studies in other provinces of Iran, the pattern of MS distribution does not seem to follow the latitude theory. Moreover, in Qom, women are probably at a higher risk of the disease, and the season and month of birth, and lack of adequate sunlight exposure may be effective in the risk of the disease.
